# A Live Video Program to Prevent Chronic Pain and Disability in At-Risk Adults With Acute Orthopedic Injuries (Toolkit for Optimal Recovery): Protocol for a Multisite Feasibility Study

**DOI:** 10.2196/28155

**Published:** 2021-04-28

**Authors:** Ana-Maria Vranceanu, Jafar Bakhshaie, Mira Reichman, James Doorley, A Rani Elwy, Cale Jacobs, Neal Chen, John Esposito, David Laverty, Paul E Matuszewski, Amirreza Fatehi, Lucy C Bowers, Mitchel Harris, David Ring

**Affiliations:** 1 Integrated Brain Health Clinical and Research Program Massachusetts General Hospital Boston, MA United States; 2 Harvard Medical School Boston, MA United States; 3 Department of Psychiatry and Human Behavior Alpert Medical School Brown University Providence, RI United States; 4 Center for Healthcare Organization and Implementation Research VA Bedford Healthcare System Bedford, MA United States; 5 Department of Orthopaedic Surgery & Sports Medicine College of Medicine University of Kentucky Lexington, KY United States; 6 Department of Orthopaedic Surgery Massachusetts General Hospital Boston, MA United States; 7 Department of Surgery and Perioperative Care Dell Medical School The University of Texas at Austin Austin, TX United States

**Keywords:** orthopedic, musculoskeletal, prevention, chronic pain, disability, intervention, video, telehealth, mobile phone

## Abstract

**Background:**

Despite the pivotal role of psychosocial factors in pain and disability after orthopedic injury, there are no evidence-based preventive interventions targeting psychosocial factors in patients with acute orthopedic injuries. We developed the first mind-body intervention focused on optimizing recovery and improving pain and disability in patients with acute orthopedic injuries who exhibit high levels of catastrophic thinking about pain and/or pain anxiety (Toolkit for Optimal Recovery [TOR] after orthopedic injury). In a pilot single-site randomized controlled trial (RCT), the TOR met a priori set benchmarks for feasibility, acceptability, and satisfaction. The next step in developing TOR is to conduct a multisite feasibility RCT to set the stage for a scientifically rigorous hybrid efficacy-effectiveness trial.

**Objective:**

The objective of this study is to conduct a rigorous multisite feasibility RCT of TOR to determine whether the intervention and study methodology meet a priori set benchmarks necessary for the successful implementation of a future multisite hybrid efficacy-effectiveness trial. In this paper, we describe the study design, manualized treatments, and specific strategies used to conduct this multisite feasibility RCT investigation.

**Methods:**

This study will be conducted at 3 geographically diverse level 1 trauma centers, anonymized as sites A, B, and C. We will conduct a multisite feasibility RCT of TOR versus the minimally enhanced usual care (MEUC) control (60 patients per site; 30 per arm) targeting a priori set feasibility benchmarks. Adult patients with acute orthopedic injuries who endorse high pain catastrophizing or pain anxiety will be recruited approximately 1-2 months after injury or surgery (baseline). Participants randomized to the TOR will receive a 4-session mind-body treatment delivered via a secure live video by trained clinical psychologists. Participants randomized to the MEUC will receive an educational booklet. Primary outcomes include feasibility of recruitment, appropriateness, feasibility of data collection, acceptability of TOR (adherence to sessions), and treatment satisfaction across all sites. We will also collect data on secondary implementation outcomes, as well as pain severity, physical and emotional function, coping skills, and adverse events. Outcomes will be assessed at baseline, posttreatment, and at the 3-month follow-up.

**Results:**

Enrollment for the RCT is estimated to begin in June 2021. The target date of completion of the feasibility RCT is April 2024. The institutional review board approval has been obtained (January 2020).

**Conclusions:**

This investigation examines the multisite feasibility of TOR administered via live videoconferencing in adult patients with acute orthopedic injuries. If feasible, the next step is a multisite, hybrid efficacy-effectiveness trial of TOR versus MEUC. Preventive psychosocial interventions can provide a new way to improve patient and provider satisfaction and decrease suffering and health care costs among patients with orthopedic injuries who are at risk for chronic pain and disability.

**International Registered Report Identifier (IRRID):**

PRR1-10.2196/28155

## Introduction

### Background

Acute orthopedic injuries, such as fractures and dislocations, are highly prevalent and costly [1-3]. Regardless of objective healing (eg, via radiography or magnetic resonance imaging), up to 50% of patients with acute orthopedic injuries will eventually develop chronic pain and disability requiring additional medical treatments, further burdening the health care system [1]. During the acute phase of orthopedic injury, psychosocial factors such as pain anxiety and catastrophizing (ie, intensely negative and inaccurate thoughts regarding pain and activity) are among the strongest predictors of pain and disability [4-8], regardless of pain severity [9], pain location [7,10], or injury type [6,11,12]. To prevent chronic pain and disability among patients with orthopedic injuries, early detection and targeted intervention focused on these psychosocial factors is crucial [13,14].

Currently, there are no evidence-based preventive interventions targeting psychological factors in patients with acute orthopedic injuries [15]. Indeed, the model of care for acute orthopedic injuries is rigidly confined to the biomedical paradigm, relying almost exclusively on surgical intervention and pain medication. Although recent attention to psychosocial factors in orthopedic populations has increased surgeons’ awareness of the clinical relevance of psychological factors [16], surgeons still report reluctance and discomfort when discussing these concerns with patients or making referrals to psychological service providers [16-18]. Typically, referrals for psychological services are made when orthopedic patients have already developed chronic pain and disability, missing the critical period for preventive intervention [17,18]. Other barriers to the provision of psychosocial care for patients with orthopedic injuries include stigma (eg, related to diagnostic labels and mental health treatment), transportation and time constraints, and lack of psychological care providers with training relevant to the orthopedic population [5,19].

Mind-body interventions tailored to address the psychosocial needs of patients with acute orthopedic injuries offer a novel approach for improving pain and disability [20-25]. Mind-body interventions have become increasingly popular in recent years among medical patients, including those with orthopedic conditions, and represent a powerful avenue for engaging patients in psychosocial care despite the stigma associated with mental health services [20-25]. In line with this trend, our group developed Toolkit for Optimal Recovery (TOR) after orthopedic injury, the first mind-body program focused on optimizing recovery and improving pain and disability-related outcomes in patients with acute orthopedic injuries who are at risk for chronic pain and disability as a function of psychological factors (ie, exhibit high levels of catastrophic thinking about pain and/or pain anxiety) [26]. Rooted in the theoretical framework of the fear avoidance model [27], TOR targets catastrophic thinking about pain (eg, misconceptions about pain and activity, hopelessness, helplessness, magnification of pain) and pain anxiety (eg, negative pain-related thoughts, fear of pain, hypervigilance, heightened physiological reactivity to pain sensations, and pain-related escape and avoidance).

**Figure 1:**
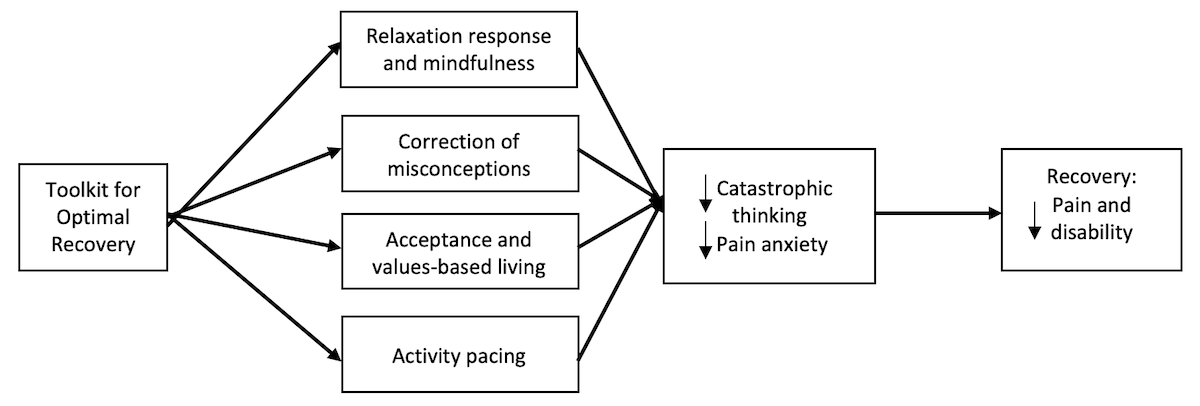
Conceptual model of Toolkit for Optimal Recovery for patients with acute musculoskeletal injury who are at risk for the development of chronic pain and disability.

TOR helps patients *confront* rather than avoid their pain-related experiences through teaching relaxation response skills (ie, exercises designed to decrease heart and breath rate) [28] and mindfulness skills (ie, intentional self-regulation of attention from moment to moment) [29], correction of misconceptions about pain and physical activity, acceptance (eg, of pain), values-based activity engagement, and activity pacing. Conceptually, participation in the TOR will be associated with decreased catastrophic thinking about pain and pain anxiety. In turn, these factors will lead to improved pain and disability-related outcomes and reduce the risk of chronic pain and disability (see Figure 1 for an illustration of this conceptual model). TOR was developed iteratively with feedback from patients, surgeons, and other orthopedic care providers and uses a telehealth delivery method with the goal of increasing access, cost-effectiveness, and service satisfaction [26].

We previously found evidence for feasibility, acceptability, and satisfaction for TOR in a pilot feasibility randomized controlled trial (RCT) conducted at a single orthopedic trauma center [26]. Despite these positive outcomes, our pilot RCT revealed several methodological weaknesses that needed to be addressed before a hybrid efficacy-effectiveness trial. In addition to generalizability concerns due to single-site sampling, half of the surgeons within the recruiting orthopedic department (2 out of the 4) did not cooperate in making referrals. Specifically, surgeons reported concerns about disrupting clinical flow within the orthopedic department and expressed skepticism regarding the clinical relevance of psychological interventions for patients with acute orthopedic injuries. These challenges were consistent with previous work demonstrating surgeons’ discomfort referring patients to psychological interventions because of factors such as low mental health literacy, lack of training, lack of confidence in the system, and fear of upsetting patients [16]. Despite surgeons’ reluctance to refer patients to our pilot RCT, we did observe that a *warm handoff* (where surgeons personally introduced research assistants [RAs] to patients) was the most effective manner to recruit and enroll participants. We now understand that buy-in from all surgeons and orthopedic staff is pivotal for successfully implementing our study and interventions. To increase buy-in, we need to educate orthopedic care teams on the relevance of psychosocial care. We also need to listen to their feedback on how to seamlessly integrate our study into clinical flow to ensure that patient care is optimized and not disrupted. The study also highlighted the importance of recruiting a racial, ethnic, and economically diverse sample to increase the generalizability of results.

To address these limitations and to maximize the success of our planned multisite feasibility RCT, we conducted focus groups with orthopedic surgeons and staff at 3 level 1 trauma centers (86 participants recruited from the 3 sites). We used elements of Proctor’s Implementation Outcomes Framework [30] and the Consolidated Framework for Implementation Research [31] to understand the (1) barriers and facilitators to integrate psychosocial care into orthopedic care broadly, (2) barriers and facilitators to implement our study interventions and procedures, and (3) feedback for the development of training materials to educate orthopedic providers regarding the importance of psychosocial factors and facilitate provider buy-in within each site. Using this method, we gathered information necessary to optimize the implementation of TOR and related study procedures using the implementation strategies of the Expert Recommendations for Implementing Change group [32], which helped refine our protocol.

### Objectives

Here, we outline the protocol for our multisite feasibility RCT (informed and refined by the findings of the focus groups), which is a necessary step in preparing for a future hybrid efficacy-effectiveness trial. The goal of this trial is to evaluate the feasibility of our study procedures and interventions (TOR and minimally enhanced usual care [MEUC]) at 3 sites based on a priori set benchmarks of acceptability, feasibility, appropriateness, and fidelity (at the provider and patient levels). Our secondary goal is to maximize the recruitment and retention of racial and ethnic minorities to achieve generalizable results. The results will inform a fully powered, multisite, hybrid efficacy-effectiveness study of TOR versus MEUC.

## Methods

### Study Design

This study will use a multisite RCT design to evaluate the feasibility of TOR delivered via secure live video, in line with the guidelines for iterative optimization of study design and methodology before conducting a fully powered, hybrid efficacy-effectiveness trial. The institutional review board (IRB) at site A approved all the study procedures. With a cross-site reliance agreement, the site A IRB and an external data safety and monitoring board will oversee the implementation of all study-related procedures at all sites.

The study protocol was informed by and refined based on the qualitative data collected during the focus groups and exit interviews. Specifically, we revised the initially proposed study protocol for the multisite RCT, including milestones, recruitment and retention procedures, and inclusion and exclusion criteria and added strategies to maximize success based on anticipated potential challenges at each site (see Multimedia Appendix 1 for peer review of this research proposal by the National Institutes of Health).

### Setting

This multisite study will be conducted at 3 geographically diverse level 1 trauma centers, anonymized as sites A, B, and C.

### Inclusion and Exclusion Criteria

Participants will be patients from sites A, B, and C, with acute orthopedic musculoskeletal injuries who are at risk for chronic pain and disability based on high pain catastrophizing or anxiety scores (see the *Assessments* section) and who meet other inclusion and exclusion criteria (Textbox 1). At each of the 3 sites, we will recruit and enroll approximately 60 patients with acute musculoskeletal injuries (1-2 months after injury or surgery; 180 participants in total), representing a target sample size in accordance with the guidelines for feasibility trials.

**Inclusion and exclusion criteria for participants in the study.**

Inclusion criteria:

- Male and female outpatients in a level 1 trauma center of one of the 3 sites, aged 18 years or above
- Sustained a single acute orthopedic injury (eg, fracture, dislocation, or rupture) approximately 1 to 2 months earlier (acute phase)
- Scored ≥20 on the Pain Catastrophizing Scale [31] or ≥40 on the Pain Anxiety Symptoms Scale–Short Form [32]
- Willing to participate and comply with the requirements of the study protocol, including randomization, questionnaire completion, and potential home practice and weekly sessions
- Free of concurrent psychotropic medication for at least 2 weeks before initiation of treatment or stable on current psychotropic medication for a minimum of 6 weeks and willing to maintain a stable dose (ie, no psychotropics or stable for >6 weeks)
- Cleared by the orthopedic surgeon for study participation
- Able to meaningfully participate (eg, speak English and have a stable living situation as determined by the medical staff at each site)

Exclusion criteria:

- Diagnosed medical illness that is expected to worsen in the next 3 months (eg, malignancy)
- Serious mental illness or instability for which hospitalization may be likely in the next 3 months
- Current suicidal ideation
- Other serious injuries that occurred alongside the orthopedic injury
- Lifetime history of schizophrenia, bipolar disorder, or other psychotic disorder
- Current substance use disorder
- Practice of yoga, meditation, or other mind-body techniques once per week for 45 minutes or more within the past 3 months
- Currently in litigation or under workman’s comp
- Surgery complications (eg, infection or need for repeat surgery)
- Self-reported pregnancy

### Identifying, Recruiting, Consent, and Randomization Procedures

The RA at each site will review the electronic medical records of the outpatient orthopedic clinics on a daily basis, or when instructed by the medical or surgical staff, to identify participants coming into the clinic who have had an injury or a surgery approximately 1 to 2 months earlier. All sites have routine follow-up appointments scheduled during this timeframe.

The RA will provide a list of potentially eligible patients to the orthopedic medical staff, and the medical assistant in charge of checking patients into rooms will inform the patients that they are potential study participants, obtain verbal consent from them to participate in the screening procedures, and ask the patients to complete the self-report measure of pain catastrophizing and pain anxiety [33,34]. If there are too many eligible patients in one day, we will prioritize racial and ethnic minorities. For patients who screen in on either of these 2 measures, the medical assistant or RA will notify the orthopedic surgeon that the patient is a potential study participant (verbally or via a note with the study logo attached to the door). Surgeons will also receive information regarding the race and ethnicity of each participant collected by the RA from the electronic medical records in order to prioritize study referrals. The orthopedic surgeon will perform the medical visit and subsequently introduce the study to the potential participant using a predetermined script and materials and procedures developed with information from the focus groups. For patients who express interest, the surgeon will conduct a *warm handoff* referral to the RA, who will finish the screening process. If patients across sites are consistently unable to stay past their scheduled appointment window to finish all baseline procedures, we will flexibly utilize an alternate recruitment procedure in which patients are screened by phone before coming in for their 1- to 2-month follow-up visit so that they can be notified in advance if they are eligible and budget time for the research visit.

If a participant meets the eligibility criteria, the RA will meet with the participant in a private location following their clinic visit to describe the study in detail, including the consent form. All patients will have the opportunity to ask questions and will be given time to consider whether to participate. Participants who choose to participate will be asked to sign the consent form. Participants will receive a copy of the consent form. Next, participants will complete the baseline assessments in the clinic and will be randomized. A trained RA will assist with performance-based measures of physical function (the grip test will be used for participants with upper extremity injuries or walk tests for participants with lower extremity injuries). Self-report measures will be completed using an iPad (Apple Inc). The RA will be available to ensure that all questions are answered and assist with completion, as needed. Participants will be compensated after baseline assessments.

After baseline assessments, participants will be randomized to TOR or MEUC in a 1:1 ratio according to a computer-generated randomization schedule. Participants randomized to the TOR will receive instructions for live video intervention before leaving the clinic. This includes (1) installing Zoom (Zoom Video Communications) and teaching participants how to use it, (2) scheduling their 4 weekly intervention sessions with the clinician, (3) setting up EZ Texting to receive reminders for sessions and home practice, and (4) installing the TOR web platform (with session content and guided exercises) as a smartphone app. Participants who do not own a smartphone will be given one, and the RA will set up their phone at the first visit. We will also provide study participants with a data plan for the duration of the study or brainstorm ways to access free internet (eg, library or friends’ houses), if needed.

The RA will provide a printed educational booklet along with access to a web-based version of the booklet content to participants randomized to the MEUC. Participants will be assisted in accessing this website from their preferred device and bookmarking the website, as desired. Participants in MEUC who do not own a smartphone will not be provided with one, as they can also access the educational information in printed form.

### TOR Program Structure

TOR is a 4-session live video–based, individual mind-body program that aims to optimize recovery and prevent persistent pain and disability. It directly targets catastrophic thinking about pain (eg, misconceptions about pain and activity, hopelessness, helplessness, and magnifications of pain) and pain anxiety (cognitive, physiological, and pain avoidance elements) by teaching relaxation response and mindfulness skills, correction of misconceptions (eg, through education and adaptive thinking techniques), acceptance and value-based engagement in activities, and activity pacing. TOR introduces and emphasizes skills through didactics, in-session activities, discussions, and home practice assignments. The latest iteration of TOR (following the pilot RCT) consists of four sessions (4 weeks; 45-minute sessions). Home practice assignments will involve practicing TOR skills. Practicing will be facilitated through a web platform that will include all program skills and instructions as individual recordings. The study staff will download this web platform on TOR participants’ phones as a web-based app after randomization. Table 1 shows all the TOR components. For more details on TOR and its components, see the report of pilot feasibility RCT in the paper by Vranceanu et al [26].

The study clinicians are 2 doctoral-level clinical psychologists based at site A, with background and clinical training in mind-body interventions and heterogeneous pain. Clinicians will deliver TOR remotely to the participants at all sites. Makeup sessions with study clinicians will be scheduled for participants who miss any session.

**Table 1:** Descriptions of the four sessions in the Toolkit for Optimal Recovery, an individual live video–based active intervention.

Session	Topics	Skills
1	Discuss treatment rationale and goals Review and correct misconceptions about recovery trajectory after orthopedic injury Normalize pain after an injury, move patients away from the mind-body dichotomy by discussing how all pain sensations originate in the brain, and discuss the difference between hurt versus harm Learn how the sympathetic nervous system influences symptoms; learn about the disability spiral and how it can lead to slower recovery and chronic pain after orthopedic injury; and learn about the physical, emotional, and cognitive factors that can speed or slow recovery after orthopedic injury Provide education about the parasympathetic nervous system and relaxation and demonstrate relaxation strategies (diaphragmatic breathing and body scan) Set goals for skills practice: practice 1 relaxation strategy daily	Pain and recovery misconceptions Relaxation response (diaphragmatic breathing and body scan)
2	Practice diaphragmatic breathing, review previous material and homework, and problem-solve barriers to practice Conduct mindfulness exercise on pain sensations; assist patients in identifying what thoughts, feelings, and behaviors are triggered by the pain sensations and normalize this experience; and provide education about mindfulness techniques for observing thoughts, feelings, and behaviors nonjudgmentally Learn decision tree for unhelpful thoughts: adaptive thinking or reframing of thoughts that are not true (eg, “Pain means that I am getting worse”) and acceptance, validation or compassion, and letting go of thoughts previously reframed that keep coming back or those that are true but not helpful (eg, “It is harder to walk right now”) Set goals for skills practice: practice diaphragmatic breathing, body scan or mindfulness on pain daily, and complete at least one decision tree exercise	Mindfulness and meditation Adaptive thinking or restructuring versus acceptance of thoughts
3	Practice diaphragmatic breathing, review previous material and home practice, and problem-solve barriers to practice Provide rationale for activity pacing, assist patients in setting activity goals consistent with their values, and normalize avoidance of activities that are associated with injury and reinforce rationale for approach Assist patients in applying acceptance and reframing or problem-solving skills to achieve activity-pacing goals Set goals for skills practice: practice diaphragmatic breathing, body scan, or mindfulness daily; complete at least one decision tree exercise, including options for problem solving, acceptance, and reframing; and follow activity-pacing protocol	Activity pacing Value-based living
4	Practice diaphragmatic breathing, review previous material and home practice, and problem-solve barriers to practice Review all skills, assist patients in identifying which skills are being used, how helpful they are, and how they can be implemented in the future Interactive quiz to identify the improvements patient has made, skills that are being used, skills the patient would like to continue to work on, and a plan for continued coping	Skill consolidation and review

### Live Video Delivery

The TOR intervention will be delivered remotely to all participants by the study team at site A. We will use a Health Insurance Portability and Accountability Act–approved secure videoconferencing software (Zoom) routinely used in clinical practice at site A to deliver the intervention. The study team has substantial experience with the implementation of similar mind-body programs through live videos [35-37]. Following randomization, the RA at each site will install the videoconferencing software (Zoom) on the TOR participants’ devices and teach the participants how to use it. We will send a reminder email to the participants for all sessions, including a link to connect to the live video session. An RA will be available to provide real-time support with any connection issues or other technical difficulties that may occur during the intervention sessions.

### Treatment Fidelity and Patients’ Safety Considerations

We will adhere to the National Institutes of Health guidelines [38] to ensure treatment fidelity. We will use a structured manual, already developed and used in our previous single-site feasibility trial. The principal investigator (PI; AMV) will train all study clinicians on the study protocol and manualize the TOR intervention. Study clinicians will complete fidelity checklists after each session and receive weekly supervision from the senior psychologist. All study sessions will be audio recorded, and 20% of the audio recordings will be randomly selected to review for fidelity. A total of 2 independent raters will assess the selected sessions against a fidelity checklist to evaluate the fidelity of intervention delivery, with attention to rater discrepancies.

### Considerations for Participant Safety During a Virtually Delivered Program

Special safety considerations must be taken to ensure participant safety, accurate data collection, and uninterrupted live video intervention delivery. Our system for virtual program delivery is similar to the current system for site A psychologists and physicians who deliver virtual patient care. As with standard outpatient clinical care at site A, we will have full support from the site A telehealth department to ensure immediate attention to any technical issues. The PI has conducted a live video pilot clinical trial for patients with acute orthopedic trauma and is currently conducting one for patients with neurofibromatosis from across the globe with no technical difficulties. The study team will also receive training in the software, including strategies for resolving potential technical issues. We will set up and test the Zoom software during the baseline visit to the clinic to ensure that technical issues during study visits are minimized. All intervention participants will be instructed on how to rejoin the study visit if the connection is disrupted. The RA will be available to assist clinicians with technology during study visits as needed (eg, helping participants rejoin the session while the clinician continues delivering material). We will closely monitor any severe psychological symptoms, including suicidality, and provide referrals for appropriate levels of care as needed. During enrollment, all intervention patients will be asked to provide names, email addresses, and phone numbers of 2 emergency contact persons for this purpose. We will inform all patients that in the case of suicidality, a warm handoff will be performed to refer patients to local psychological services for a safety evaluation. In the unlikely event of serious concerns of suicidality and to ensure patient safety, we will suspend confidentiality and alert the site PIs so that appropriate clinical interventions can be assured. Safety will always be prioritized over participation in the study.

### The MEUC Control

Participants in the MEUC will receive a booklet containing brief, summarized information that reflects the active intervention topics, including the trajectory of pain and recovery after orthopedic illness, the role of relaxation strategies to manage pain, and the importance of returning to engagement in activities of daily living. In addition, similar to participants in the TOR group, participants in this group will receive usual medical care as determined by the medical team. Usual care involves meetings with surgeons, medical staff, pain medications, and physical therapy. Usual care is identical in the intervention and control groups.

### Protocol Fidelity and Investigator Compliance

The PI will lead a half-day session with the teams at each of the 3 sites to review the study protocol. The PI will also lead weekly research team meetings with all sites to review study operations and compliance (eg, screening, recruitment, data collection, and management), discuss any emerging concerns, identify training needs, and address any human subject issues. At the end of each study year, the designated Data and Safety Monitoring Committee will audit cases of 5 randomly selected participants and review compliance with study activities at all sites as per the study protocol.

### Assessments

We selected the measures and assessment domains in line with the Initiative on Methods, Measurement, and Pain Assessment in Clinical Trials and Outcome Measures in Rheumatology guidelines [39], study aims, and guidelines for feasibility trials [40]. All assessments presented below except those indicated otherwise will be collected at baseline (preintervention), postintervention, and at a follow-up timepoint of 3 months.

#### Sociodemographic Information

We will collect data on age, gender, race, ethnicity, education, employment, income, and marital status using a demographic questionnaire. We will also collect information on mental health history, current psychotropic or pain medication intake, substance use history and status, comorbid medical conditions, and history of depression or other mental health conditions. We will also collect data on smoking status, alcohol use, marijuana intake, and narcotic and nonnarcotic analgesic intake. These assessments will only be conducted at baseline.

#### Clinical Variables

We will collect data on the injury type, date and location, and injury severity (Abbreviated Injury Severity Index) [41] as rated by the surgeon. These assessments will only be conducted at baseline.

### Primary Outcomes

#### Feasibility of Recruitment

We will calculate the percentage of participants who agree to participate from those who are approached. We will also calculate the subgroup of minorities in each group.

#### Appropriateness of Treatment

The Credibility and Expectancy Questionnaire [42] assesses treatment expectancy and credibility in clinical outcome studies. This will be completed by participants randomized to the TOR after the delivery of the first session of the intervention.

#### Feasibility of Data Collection (Self-Report)

We will calculate the percentage of participants who complete the measures at the 3 timepoints (baseline, posttreatment, and 3-month follow-up).

#### Acceptability of TOR (Adherence to Sessions)

We will report the percentage of participants who are randomized within 1 arm and complete the posttest.

#### Treatment Satisfaction

The Client Satisfaction Scale [43] assesses participant satisfaction with TOR at posttreatment.

### Secondary Outcomes

#### Feasibility of Randomization or Adherence to the Assigned Arm

We will report the percentage of enrolled participants who start within 1 arm and complete posttest assessments.

#### Fidelity to Study Procedures

We will report the number of protocol deviations observed per site among all the enrolled participants.

#### Adherence to TOR Homework

We will calculate the ratio of homework logs returned and the number of days in the last week homework was practiced among patients randomized to the TOR (daily self-report log).

#### Acceptability as Rated by Therapist

We will report the percentage of participants who score over 7 on the therapist rating of participation quality in each intervention session among patients randomized to the TOR.

#### Feasibility of Data Collection for Performance-Based Measures of Physical Function

We will report the percentage of enrolled participants who complete performance-based measures of physical function (walk or grip test) at each of the 3 timepoints.

#### Feasibility of Data Collection for Rescue Analgesics (Nonnarcotic)

We will calculate the percentage of enrolled participants with complete data on nonnarcotic rescue analgesics.

#### Feasibility of Data Collection for Rescue Analgesics (Narcotic)

We will calculate the percentage of all participants with complete data on narcotic rescue analgesics.

#### Feasibility of Data Collection for Adverse Events

We will calculate the percentage of enrolled participants with complete data on adverse events (see information on adverse events data collection under the *Adverse Events* section).

#### Therapist-Rated Adherence or Fidelity of the Participants to Session

We will calculate the percentage of participants who present ≥75% adherence, indexed by the study clinician using checklists and audio recordings at each intervention session.

#### Pain

##### The Numerical Rating Scale

The Numerical Rating Scale (NRS) is a commonly used measure of pain intensity that has demonstrated good psychometric properties [44]. The item assesses pain using an 11-point scale ranging from *no pain* (0) to *worst pain imaginable* (10). This measure will be used to assess pain during rest and activity. We will report the sensitivity to detect changes in pain as determined by the percent change in NRS score.

##### Within-Group Change in Rescue Analgesics (Narcotic and Nonnarcotic)

We will calculate the changes in narcotic and nonnarcotic analgesics and concomitant pain treatment (daily self-report log).

#### Self-Reported Physical Function

##### Patient-Reported Outcomes Measurement Information System–Physical Function (Version 1.0)

We will calculate sensitivity to detect changes in self-reported physical function as determined by percent change. This questionnaire assesses one’s ability to perform activities that require physical actions, ranging from low-impact tasks (eg, self-care, bathing, and dressing) to vigorous physical activities (eg, running, strenuous sports). The questionnaire consists of 121 Likert scale items, with response options ranging from 1 (*without any difficulty* or *not at all*) to 5 (*unable to do*). The items do not refer to a particular recall period but rather involve the participant’s status at the time of completion [45]. The resulting *t* score is a standardized score with a mean of 50 (SD 10). Lower scores are indicative of greater disability.

##### The Short Musculoskeletal Function Assessment Questionnaire

We will report sensitivity to detect changes in self-reported physical function as determined by percent change. This questionnaire is a validated 46-item survey that measures physical functioning and musculoskeletal disability [46]. It was developed from a 101-item parent questionnaire with excellent psychometric properties [47]. The individual items are rated on a 4-point Likert scale, with high scores indicative of higher disability. It consists of 2 subscales calculated by summing up the individual items: (1) assessment of function (34 questions) and (2) perception of bothersomeness of the symptoms (12 questions). These sum scores will be transformed to final scores ranging from 0 to 100.

##### Performance-Based Physical Function (Walk or Grip Test)

We will calculate the sensitivity to detect changes in performance-based physical function by percent change. For those with lower extremity or body injuries, we will conduct a timed 10-m walk test [48]. This test is a valid assessment of walking speed when used as a time indicator of health status. The participants will be asked to walk without assistance for 10 m. The time will be measured for the intermediate 6 m, allowing for acceleration and deceleration. Participants will be permitted to use assistive devices as long as they are kept consistent and documented. For those with upper extremity or body injuries, a grip strength test [49] will be conducted. This test is a commonly used simple measure of muscle strength. The strength of the participants’ grip will be measured quantitatively using a hand dynamometer [49].

#### Coping and Emotional Function

We calculated sensitivity to detect changes in coping and emotional function, as determined by the percent change in these outcomes using the following measures.

##### The Pain Catastrophizing Scale

The Pain Catastrophizing Scale (PCS) is a measure of negative pain-related cognitions or catastrophic thinking (exaggerated reporting and rumination about pain), which has demonstrated good psychometric properties [33]. The PCS includes 13 Likert scale items with 4 points ranging from *not at all* (0) to *all the time* (3). The PCS consists of 3 subscales: (1) rumination (ie, repetitively going over thoughts and feelings about pain), (2) helplessness (ie, subjective feeling of hopelessness or helplessness related to the experience of pain), and (3) magnification (ie, exaggerated thinking about the negative consequences of pain). A total score will be computed, with higher scores reflecting poorer coping ability. A score ≥20 on PCS has been proposed as an indicator of high psychosocial risk for poor outcomes after orthopedic trauma and will be used as one of the inclusion criteria in this trial [50]. PCS will be administered before every assessment and intervention session.

##### The Pain Anxiety Symptom Scale 

The Pain Anxiety Symptom Scale (PASS-20) [34] is a measure of fear and anxiety related to the experience of pain. PASS-20 consists of 20 items rated on a 6-point Likert scale from *never* (0) to *always* (5). The PASS-20 consists of 4 subscales: (1) avoidance (ie, avoiding activities that cause pain), (2) fearful thinking (ie, fear thoughts related to pain), (3) cognitive anxiety (ie, compromised cognitive function when in pain), and (4) physiological response (ie, somatic anxiety symptoms in response to pain). A total score will also be computed, with higher scores reflecting poorer coping ability. A score ≥40 on the PASS-20 is proposed as an indicator of high psychosocial risk for poor outcomes after orthopedic trauma and will be used as an inclusion criterion in this trial [51,52]. PASS-20 will be administered before every assessment and intervention session.

##### Measures of Current Status

The Measures of Current Status (MOCS) [53] assesses the perceived ability to use healthy coping skills. The MOCS consists of 4 subscales: (1) relaxation, (2) awareness of stress, (3) assertiveness, and (4) disputing maladaptive thoughts. The MOCS consists of 13 items rated on a 5-point Likert scale ranging from 0 (*I cannot do this at all*) to 4 (*I can do this extremely well*). A total score will be computed with scores ranging from 0 to 52, with higher scores reflecting more effective coping ability.

##### The Posttraumatic Stress Disorder Checklist: Civilian Version

The Posttraumatic Stress Disorder Checklist (PCL) is a measure of posttraumatic stress symptoms with good psychometric properties [54]. The PCL consists of 17 items rated on a 5-point Likert scale ranging from 0 (*not at all*) to 4 (*all the time*). The items measure the extent to which the participant has been bothered by symptoms of posttraumatic stress in the past month. The measure provides a total severity score as well as a diagnostic cutoff.

##### The Center for Epidemiologic Study of Depression 

The Center for Epidemiologic Study of Depression [55] is a commonly used measure of depression symptoms. It consists of 20 items rated on a 4-point Likert scale from *rarely or none of the time (less than 1 day)* (0) to *most or all the time (5-7 days*). The measure provides a total severity score for depressive symptoms.

##### Adverse Events

We will calculate the number of adverse events among all enrolled participants (data collected at each assessment and intervention timepoints).

##### Feasibility of Collecting Orthopedic Staff Satisfaction Measures

We will calculate the percentage of surgeons and study staff who complete the measures related to staff satisfaction with the study procedures.

##### Feasibility of Obtaining Data on Orthopedic Staff Perceived Ease of Referrals

We will report the percentage of surgeons and study staff who complete the measure.

##### Feasibility of Obtaining Data on Orthopedic Staff Perceived Cost-Benefit

We will report the percentage of surgeons and study staff who complete the measure.

##### Orthopedic Staff Feasibility of Referral

We will calculate the percentage of surgeons who make at least 5 referrals per site.

##### Feasibility of Obtaining Data on Feasibility of Study Implementation From Staff

We will calculate the percentage of surgeons and staff who complete the data.

##### Feasibility of Collecting Appropriateness Measures by Staff

We will report the percentage of surgeons and staff who complete appropriateness measure.

##### Perceived Acceptability of Study by Staff

We will report the percentage of surgeons and staff who score over 7 on a 10-item Likert scale satisfaction measure, among those who completed the measure (collected at the end of the study).

##### Perceived Acceptability of Staff Regarding Ease of Referral

We will calculate the percentage of surgeons and staff who score over 7 on a 10-item Likert scale ease of referral measure, among those who completed the measure (collected at the end of the study).

##### Perceived Acceptability of Staff Regarding Cost-Benefit

We will calculate the percentage of surgeons and staff who score over 7 on a 10-item Likert scale cost-benefit measure, among those who completed the measure (collected at the end of the study).

##### Feasibility of Study Implementation as Perceived by Study Staff

We will calculate the percentage of surgeons and staff who score over 7 on a 10-item Likert scale of feasibility of study implementation as perceived by study staff, among those who completed the measure (collected at the end of the study).

##### Appropriateness as Perceived by Study Staff

We will calculate the percentage of surgeons and staff who score over 7 on a 10-item Likert scale of appropriateness, among those who completed the measure (collected at the end of the study).

### Analysis Plan and Sample Size Considerations

We will evaluate the feasibility of each site based on the number of patients identified via medical records, the number referred by the surgeon, number screened, number consented, and number enrolled and randomized. The results will be reported using descriptive statistics (ie, numbers and proportions with 95% CI).

The target sample size of 60 participants enrolled per site (180 participants in total) will provide sufficient data to evaluate the feasibility benchmarks required for future hybrid efficacy-effectiveness studies. With 1:1 randomization, we will have 30 participants per arm, which is the minimum required to adequately evaluate measures of fidelity, acceptability, appropriateness, and feasibility. This trial is neither powered for efficacy nor aimed to provide such information. Consistent with the feasibility design of this trial, we will report the means and SDs of all measures at all timepoints, including the distribution of scores and internal consistency. To determine the measures’ sensitivity to detect changes, we will report the percent change in all quantitative outcomes. We will also summarize demographic and clinical variables. Although we will not conduct any efficacy analyses consistent with previous research recommendations [40], we will use estimates of person-to-person variation in efficacy outcomes to determine the required sample size for a future hybrid efficacy-effectiveness trial.

The following benchmarks will be required to be met to proceed to implement a fully powered RCT:

- Overall, ≥70% of patients approached will have to agree to participate (feasibility of recruitment).
- Among all surgeons and study staff: ≥70% of staff will provide data on the feasibility and appropriateness of study implementation, staff satisfaction with study procedures, perceived ease of referral, and cost-benefit. In addition, to achieve feasibility of referral, ≥80% of surgeons need to make at least 5 referrals per site.
- Among surgeons and study staff who complete measures: ≥70% of staff will endorse a score of over 7 on acceptability, defined as staff satisfaction with study procedures, perceived ease of referral and cost-benefit, and feasibility and appropriateness of study implementation.
- Among all patients with orthopedic injuries: ≥70% who were approached agreed to participate, with <5 protocol deviations per site.
- Among all enrolled patients with orthopedic injuries: ≥70% of participants will have to complete self-report data at each of the 3 timepoints; ≥70% of participants who start within one arm must complete posttest; ≥70% of patients must agree to complete walk tests and grip tests; and ≥70% of participants will provide data on narcotic and nonnarcotic analgesics and adverse events, and we will observe stability of nonnarcotic analgesics, decrease in narcotic analgesics, and minimal adverse effect during the study period.
- Among patients randomized to TOR: ≥70% of participants will endorse a score over the scale’s midpoint on credibility and expectancy score and client satisfaction score, ≥3 out of 4 sessions attended by ≥70% of participants, ≥1 out of 3 homework logs returned with 4 out of 7 days of practice by ≥70% of participants, and ≥75% of sessions with a score over 7 on the therapist rating of participation quality.
- We will observe ≥75% adherence by the therapist (indexed through checklist and audio recordings) for ≥70% of the participants.

Once these data analyses are completed, the multidisciplinary team will review the data and discuss the interpretation of our findings in the context of current research on acute orthopedic injury-related pain and disability. In the event that a given benchmark is not met based on the preliminary data collected, site-specific modifications will be made.

### Data Management

The research team will develop standard operating procedures for data collection, data management, and quality control. The PI (along with 2 co-PIs) will oversee all procedures related to secure data collection and management. Data activities will be conducted using Research Electronic Data Capture (REDCap), a software and workflow tool for research data collection and management, with assistance from the Partners HealthCare Research Computing, Enterprise Research Infrastructure and Services group. REDCap provides a secure web-based app with intuitive user interface data and real-time validation rules. All data will be stored in REDCap at site A.

### Data Quality

Per study year, the data safety and monitoring committee will receive a data quality report for each site and per randomized group, including a summary of (1) adverse events per randomized group, including the number and types of events, severity, timepoint, and determination of whether the events were study related; (2) an unblinded report of study retention and reasons for attrition per randomized group; and (3) a data quality report for each site, including a summary of participant enrollment and retention, participant adherence with visits, and data completeness and quality. The committee will use this report to evaluate the study’s capacity for valid analysis.

### RCT Deliverables

We will report on feasibility outcomes for TOR delivery at each site, including the ability to recruit diverse racial, ethnic, and low socioeconomic status populations. We will also obtain the required sample size for a future hybrid efficacy-effectiveness trial.

## Results

We have initiated the pre-enrollment procedures for the feasibility RCT at each site, with enrollment set to begin in June 2021. The target date of completion for the feasibility RCT is April 2024.

## Discussion

### Principal Findings

This paper describes the study design and specific strategies used to conduct a multisite feasibility RCT of TOR administered via live videoconferencing to adult patients with acute orthopedic injuries across 3 sites. We provide details on assessing the feasibility of the refined program at 3 heterogeneous level 1 trauma centers that involve demographically diverse patients and implement different guidelines and routines, including overcoming typical barriers to psychosocial care by delivering the intervention via a secure live video. This information is invaluable for future research and provides a novel paradigm for the delivery of preventive care to patients with acute orthopedic injuries.

The long-term goal of this project is to set the stage for a multisite hybrid efficacy-effectiveness trial of TOR versus MEUC. Informed and refined by the findings of the focus groups conducted among orthopedic medical provider stakeholders, the results of this study will address the challenges concerning barriers to biopsychosocial care for orthopedic patients using an implementation framework. Thus, in addition to the use of novel methods such as delivering the intervention via secure live video, we also revised the initially proposed study protocol (mainly recruitment strategy and inclusion and exclusion criteria) and developed educational materials for the care team based on focus group data to maximize success at each site. We will also be able to address some limitations of the single-site feasibility RCT, such as the lack of ethnic, racial, and socioeconomic diversity in the study population. This line of work has the potential for adaptation and generalizability to surgical orthopedic centers and even primary care practices.

The findings will also provide a realistic examination of the study methods, as they will take place in a fully powered, hybrid efficacy-effectiveness trial. Specifically, by seeking feedback from providers and staff during the commencement of this study, we have gained knowledge regarding the potential site-specific differences that need to be addressed for future hybrid efficacy-effectiveness trials and dissemination of our effective intervention. Our efforts to refine the TOR program based on this feedback and achieve a priori feasibility benchmarks of this study will allow for the successful implementation of a scientifically rigorous multisite hybrid efficacy-effectiveness trial. Our guiding hypotheses for this next trial are that receiving TOR delivered over live video at 1 to 2 months after an orthopedic injury will be associated with improvements in pain and disability, catastrophic thinking about pain, pain anxiety, and depressive and posttraumatic symptoms, compared with MEUC. Using the findings of this hybrid efficacy-effectiveness trial, we aim to set the stage for dissemination studies.

### Foreseen Challenges

Despite the innovative approach of this project, some potential challenges warrant consideration. First, the recruitment of ethnically diverse samples could be challenging. The 3 sites of this study are geographically dispersed and encompass a diverse sociodemographic sample. We will also approach every ethnic and racial minority participant and prioritize their recruitment (see the Methods section). If by the end of year 2, we do not end up with at least 25% minorities in our study samples across sites, we will expand our recruitment to a preidentified urban medical center with 80% racial and ethnic minority patients. Second, there are concerns regarding the competing research projects. To overcome this barrier, we have secured support from orthopedic leaders at each site to prioritize this study at each site. Third, special considerations exist regarding live video interventions in terms of participants’ safety, accurate data collection, and uninterrupted delivery of the live video intervention. In addition to having the full support of site A telehealth, we have ensured that our study team has adequate training in patient safety considerations, use of the software, and problem solving of any potential technical issues.

### Implications

The recognition of the pivotal role of psychosocial interventions in orthopedic care is increasing. However, this awareness has yet to be reflected in the development of feasible psychosocial interventions to address this need or in real-life practices of the surgeons and related medical staff who do not reliably refer their patients to receive psychosocial support. This multisite feasibility RCT is a part of the work toward developing the first preventive psychosocial intervention to address the development of chronic pain and disability among at-risk patients with acute orthopedic injuries. The results will inform the fully powered hybrid efficacy-effectiveness trial, which will utilize the benchmarks and program refinements established by this study. The goal is to improve the care of orthopedic patients with musculoskeletal injuries who are at risk for chronic pain and disability, by shifting the paradigm of care for these patients from a purely medical model to a biopsychosocial model with provision of psychosocial interventions that are acceptable (by the patients and care team) and easily delivered within all orthopedic trauma practices. Such a paradigm shift will provide a new way to improve patient and provider satisfaction, decrease suffering, and decrease health care costs among this group of patients. Moreover, if effective, this model of care and related implementation framework can be applied in other medical settings.

## Appendix

Multimedia Appendix 1 Peer-review report by the National Center for Complementary and Integrative Health Special Emphasis Panel, Exploratory Clinical Trials of Mind and Body Interventions (National Institutes of Health).

## Abbreviations

IRB: institutional review board
MEUC: minimally enhanced usual care
MOCS: Measures of Current Status
NRS: Numerical Rating Scale
PASS-20: Pain Anxiety Symptom Scale
PCL: Posttraumatic Stress Disorder Checklist
PCS: Pain Catastrophizing Scale
PI: principal investigator
RA: research assistant
RCT: randomized controlled trial
REDCap: Research Electronic Data Capture
TOR: Toolkit for Optimal Recovery
