# Supplementary material for: A Live Video Program to Prevent Chronic Pain and Disability in At-Risk Adults With Acute Orthopedic Injuries (Toolkit for Optimal Recovery): Protocol for a Multisite Feasibility Study
Source: JMIR Res Protoc. 2021 Apr 28;10(4):e28155. doi: 10.2196/28155 (PMC8116990; doi:10.2196/28155)
Supplement: Multimedia Appendix 1 [file resprot_v10i4e28155_app1.pdf]

SUMMARY STATEMENT

PROGRAM CONTACT:
Dr. Della White
301-827-6358
whitede@mail.nih.gov

(Privileged Communication)

Release Date: **12/10/2019**
Revised Date:

Application Number: **1 U01 AT010462-01A1**

Principal Investigator

VRANCEANU, ANA-MARIA

Applicant Organization: MASSACHUSETTS GENERAL HOSPITAL

Review Group: **ZAT1 PJ (07)**
National Center for Complementary and Integrative Health Special Emphasis Panel Exploratory Clinical Trials of Mind and Body Interventions (MB)

Meeting Date: **12/03/2019**
Council: **JAN 2020**
Requested Start: **04/01/2020**
RFA/PA: **PAR18-118**
PCC: **MUDDL**

Project Title: **Toolkit for Optimal Recovery after Orthopedic Injury; A multi-site feasibility study to prevent persistent pain and disability**
SRG Action: **Impact Score:23**
Next Steps: **Visit https://grants.nih.gov/grants/next_steps.htm**
Human Subjects: 30-Human subjects involved - Certified, no SRG concerns
Animal Subjects: 10-No live vertebrate animals involved for competing appl.
Gender: 1A-Both genders, scientifically acceptable
Minority: 1A-Minorities and non-minorities, scientifically acceptable
Age: 3A-No children included, scientifically acceptable

Project Year	Direct Costs Requested	Estimated Total Cost
1	462,230	764,514
2	455,299	753,050
3	455,553	753,470
4	454,124	751,107
TOTAL	1,827,206	3,022,140

ADMINISTRATIVE BUDGET NOTE: The budget shown is the requested budget and has not been adjusted to reflect any recommendations made by reviewers. If an award is planned, the costs will be calculated by Institute grants management staff based on the recommendations outlined below in the COMMITTEE BUDGET RECOMMENDATIONS section.
EARLY STAGE INVESTIGATOR
NEW INVESTIGATOR

1U01AT010462-01A1 Vranceanu, Ana-Maria

EARLY STAGE INVESTIGATOR
NEW INVESTIGATOR

RESUME AND SUMMARY OF DISCUSSION: This U01 resubmission titled "Toolkit for Optimal Recovery (TOR) after Orthopedic Injury; A multi-site feasibility study to prevent persistent pain and disability" is submitted in response to PAR-18-118 "NCCIH Mind and Body Clinical Trial Cooperative Agreement (U01)" by Massachusetts General Hospital with Dr. Ana-Maria Vranceanu as the principal investigator. The overall goal of this U01 is to assess implementation barriers and facilitators using focus groups and to conduct a multi-site feasibility RCT across three Level 1 trauma centers at Massachusetts General Hospital (MGH, Boston, MA), Dell Medical Center (Austin, TX) and Kentucky Medical Center (Lexington, KY). Specifically, the project will evaluate the feasibility and acceptability of TOR, a live video intervention targeting psychosocial factors for patients recovering from acute musculoskeletal injuries in an orthopedic setting. If successful, the project could set the stage for a future multi-site hybrid efficacy-effectiveness study to examine a novel approach focusing on psychosocial factors to mitigate the risk of long-term pain and disability, thus challenging current clinical practice. The project leverages robust preliminary data from a prior feasibility trial to inform the current study design. The investigative team are recognized leaders in their respective fields, have a history of collaboration, and have complementary multi-disciplinary expertise for the conduct of this work. Overall, the environment is strong, with adequate resources and recruitment capabilities. The panel highlighted the intervention targeting psychosocial factors to prevent the transition from acute to chronic pain for patients recovering from orthopedic traumatic injuries as innovative. The panel agreed the application was responsive to previous critiques, including the addition of an implementation scientist. The project has several notable strengths in the approach, including a strong rationale for the intervention and comparison group, clear feasibility benchmarks, and the focus on obtaining clinician buy-in. The panel remarked on minor concerns in the approach. The underlying rationale for delivering the intervention on a one-on-one basis, potentially straining limited resources, instead of by group format was not entirely clear. The rationale for employing a four-session program versus other potential options was not entirely well-justified. These minor concerns did not significantly reduce enthusiasm for the project's potential for high impact. Overall, the panel agreed that the project was highly significant, the study team and environment were highly qualified, and the development of a promising intervention could provide a novel therapeutic approach for managing psychosocial determinants of chronic pain during recovery from musculoskeletal injuries, thereby mitigating a significant public health concern.

DESCRIPTION (provided by applicant):
Using the NCCIH U01 mechanism, we aim to establish benchmarks to allow for successful implementation of a future scientifically rigorous multi-site hybrid efficacy-effectiveness trial (UG3/UH3 mechanism) of the live video, individual mind body program Toolkit for Optimal Recovery after Injury (TOR) versus a minimally enhanced usual care control (MEUC) delivered to individual patients with acute orthopedic musculoskeletal injuries at risk for chronic pain and disability. Our pilot feasibility trial at the Massachusetts General Hospital (MGH) Level 1 Trauma Clinic provided evidence for feasibility, acceptability, signal of improvement after TOR participation, and allowed us to identify limitations and barriers to recruitment and study implementations, which informed the current proposal. The multi-site feasibility study will be conducted at 3 geographically diverse Level 1 trauma centers: MGH (MA), Dell Medical School (TX), Lexington (KY). First, using elements of Proctors' Implementation Outcomes Framework we will conduct focus groups with orthopedic surgeons and staff at each site to understand barriers and facilitators to psychological care and study referrals/procedures and develop training materials to bypass these barriers and ensure integration of study procedures within each site (aim 1). Next, we will conduct a feasibility multi-site randomized controlled trial of the TOR against UC (60 patients/site; 30/arm; 50 completers) targeting a priori set benchmarks. Our team is uniquely positioned for this U01, with a psychologist (Vranceanu; overall PI) and orthopedic surgeon (Ring; Dell site PI) with

over 12 years' collaboration. The PI and statistician have extensive expertise in intervention development, feasibility testing, clinical trials, and live video intervention delivery. We recruited an implementation science expert with prior experience working in orthopedic surgery. There is excitement and support from each of the Orthopedic Trauma Units, and we recruited orthopedic surgeons passionate about psychosocial care to serve as champions at each site. We hope to enhance the care of orthopedic trauma patients through a low burden mind body program (TOR) focused on optimizing recovery and preventing chronic pain and disability. This early focus on study implementation will bolster the rigor of the subsequent UG3/UH3 trial as well as inform future TOR implementation, should this be supported by efficacy data.

PUBLIC HEALTH RELEVANCE

The U01 proposal will set the stage for a scientifically rigorous multi-site hybrid efficacy-effectiveness trial via the UG3/UH3 NCCIH mechanism. The ultimate goal is to enhance the care of orthopedic patients with musculoskeletal injuries who are at risk for chronic pain and disability, through implementation of the mind body program Toolkit for Optimal Recovery after Injury (TOR) delivered via secure live video within all orthopedic trauma practices. There are ample opportunities to improve patient and provider satisfaction, decrease suffering and health care costs.

CRITIQUE 1

Significance: 2
Investigator(s): 2
Innovation: 2
Approach: 2
Environment: 2

Overall Impact: This is an excellent application that proposes to conduct a feasibility trial at three geographically diverse level 1 trauma centers in Massachusetts, Kentucky, and Texas. Specific comprehensive benchmarks were described to allow for successful implementation of a planned future larger multisite efficacy/effectiveness trial to establish if a live video individual mind-body program for optimal recovery after a traumatic musculoskeletal injury is superior to a minimally enhanced usual care control. There are currently no established evidence-based psychosocial interventions to mitigate the risk of long-term pain and disability following the recovery of acute orthopedic traumatic injuries. The proposed mind-body program appropriately challenges the current biomedical model that informs the management of acute orthopedic injuries. The investigative leadership team is collectively highly experienced and well-suited to carry out this project, which has excellent potential for improving clinical practice if later shown to be effective. There is a good rationale for the choice of comparison group.

1. Significance:
Strengths

- The project addresses a prevalent and costly problem of the risk of psychosocial complications after biological recovery from fractures and locations. Up to half of the patients go on to develop long-term pain and disability.
- A recent systematic review by the research team showed that no evidence-based treatments are available to address what appears to be psychosocial complications.
- The proposed project is an important and timely feasibility trial of a mind-body program to optimize recovery after acute traumatic musculoskeletal injuries and prevent long-term pain and disability.
- This multisite feasibility trial is necessary to prepare for the next step of a large phase 3 trial.
- If the future planned multisite trial shows that the mind-body program is effective it could have a major public health impact.

Weaknesses
- No major weaknesses noted.

2. Investigator(s):
Strengths
- The investigative leadership team is collectively highly experienced and well-suited to carry out this project.
- The principal investigator, Dr. Vranceanu, has published over 130 articles and book chapters and has been principal investigator on 12 federal and foundation grants, of which 8 test mind and body interventions in RCTs. She has extensive experience in qualitative and quantitative analyses, intervention development, and testing. She is extremely well qualified to lead this project.
- The co-investigator, Dr. Macklin, has collaborated with Dr. Vranceanu on several previous trials assessing the effect of mind-body interventions that address psychosocial problems. He appears well-qualified for this project.
- The second co-investigator, Dr. Harris, has many years of clinical and research experience with the orthopedic injury patients.
- The rest of the project team at all three performance sites appear well-qualified.

Weaknesses
- Collectively the investigative team has some experience recruiting RCT participants from multiples sites but has limited experience with conducting multisite RCTs.

3. Innovation:
Strengths
- There are currently no established evidence-based psychosocial interventions to mitigate the risk of long-term pain and disability following the recovery of acute orthopedic traumatic injuries.
- The proposed mind-body program challenges the current biomedical model that informs the management of acute orthopedic injuries.
- The project is testing a program that addresses the needs of patients from a more comprehensive biopsychosocial perspective.
- The project has the potential for improving clinical practice.

Weaknesses
- No major weaknesses noted.

4. Approach:
Strengths
- The response to previous review recommendations are relevant and appear adequate.
- The project is informed by a well-designed and successful initial feasibility trial at a single site.
- The initial feasibility trial has already been published in a peer-reviewed journal.
- The investigative team has published a prospective study with follow-up on psychosocial factors that predict disability of pain intensity after musculoskeletal trauma.
- The investigative team has also published a systematic review of psychosocial factors in orthopedic trauma.
- The addition of an implementation scientist on the team is a major strength as is the plan identifying barriers or facilitators for study implementation through relevant focus groups and qualitative analysis.
- The description of the theoretical framework and the conceptual model informing the mind-body intervention is a clear strength.
- A comprehensive plan is in place to deliver the experimental intervention, and to assess fidelity, acceptability and adherence to study protocols.
- There is a good rationale for choice of comparison group.

- Data management of statistical analyses are clearly addressed and there is no attempt to look at between-group differences in this feasibility trial.
- The recruitment plan and the necessary refinement based on the first feasibility trial is clearly articulated.
- Feasibility and clinical outcome measures are well chosen and described.
- Relevant benchmarks for judging the readiness to move to the fully powered multisite trial are provided.
- There are strong letters of institutional support from all three trial performance sites.

Weaknesses

- There is no clear explanation for or discussion of why patients can't be recruited directly without being referred by site surgeons, nurse and other medical staff.

5. Environment:

Strengths

- The three sites that will be conducting the feasibility trial have excellent environments.
- Institutional support is adequate, and the project has a high probability of succeeding.

Weaknesses

- No major weaknesses noted.

Milestones:

Acceptable

- The benchmarks are detailed, relevant, measurable and if met will ensure the success of the proposed study

Data and Safety Monitoring:

Acceptable

- Comprehensive and well described.

Study Timeline:

Strengths

- There are well defined timelines for all four years.
- Plans for dealing with enrollment shortfalls and other likely challenges are included.

Weaknesses

- No major weaknesses noted.

Protections for Human Subjects:

Acceptable Risks and/or Adequate Protections

- Thoroughly described.

Data and Safety Monitoring Plan (Applicable for Clinical Trials Only):

- Acceptable
    - Comprehensive and described in detail.

Inclusion Plans:

- Sex/Gender: Distribution justified scientifically.
- Race/Ethnicity: Distribution justified scientifically.
- For NIH-Defined Phase III trials, Plans for valid design and analysis: Not Applicable.
- Inclusion/Exclusion Based on Age: Distribution justified scientifically.
- The choice of age range was reasonable but there was no justification for the upper limit of 70.

Vertebrate Animals:

Not Applicable (No Vertebrate Animals)

Biohazards:
Not Applicable (No Biohazards)

Resubmission:
Acceptable

Applications from Foreign Organizations:
Not Applicable (No Foreign Organization)

Select Agent Research:
Not Applicable (No Relevant Resources)

Resource Sharing Plans:
Unacceptable

- Not addressed in the application

Authentication of Key Biological and/or Chemical Resources:
Not Applicable (No Relevant Resources)

Budget and Period of Support:
Recommend as Requested

CRITIQUE 2

Significance: 3
Investigator(s): 1
Innovation: 2
Approach: 3
Environment: 2

Overall Impact: This is a U01 intended to test feasibility and acceptability of a psychosocial intervention, Toolkit for Optimal Recovery after Injury (TOR), to prevent the transition from acute to chronic pain for patients with musculoskeletal injuries (MI) treated by orthopedic surgeons. The goal is to prepare for a future multi-site hybrid effectiveness/efficacy study of the intervention. Strengths include that the intervention itself is largely developed but much more information is needed on how to implement it in settings that are not familiar with, and perhaps undervalue, such interventions. The team is excellent and was bolstered in this resubmission application by the addition of an implementation scientist. The approach to testing feasibility for a large trial is solid and will inform subsequent studies. The most prominent, although minor, weakness is that individual psychosocial interventions strain limited resources where group interventions might have greater reach.

1. Significance:
Strengths

- The foundational logic related to the type of intervention, the mode of delivery, and the broad patient phenotype is solid.
- New preliminary data were available for this application.
- Knowledge gained about how to implement this type of intervention in a surgical setting is critical to future trials and real-world implementation.
- If successful, future trials would have a high likelihood of changing clinical practice.

Weaknesses

- The rationale for including a heterogenous group of patients in terms of their site of injury, and a less heterogeneous group in terms of responses to key pre-screening psychological measures, is good, but some additional consideration of the psychological phenotype is warranted. At the very least, knowing whether a person has a prior history of being successfully treated for MI by cognitive-behavioral therapy seems important.

2. Investigator(s):

Strengths

- This is a very strong team with multidisciplinary expertise in psychology, surgery, implementation science, and biostatistics.

Weaknesses

- No major weaknesses noted.

3. Innovation:

Strengths

- The focus on working with the medical care teams and the clinics to increase feasibility with an eye toward implementation is excellent.
- Given the reluctance of prior surgeons to engage with TOR, the focus groups with surgeons and trainees could improve medical education if these groups remain a part of implementation of TOR following a successful RCT.
- Addressing psychosocial factors in these injuries is very important.

Weaknesses

- The intervention itself is not novel but that is minor.

4. Approach:

Strengths

- The design is appropriate, and the investigators were responsive to prior critiques.
- There is a relatively clear “this isn’t working” benchmark to cover that possibility.

Weaknesses

- One-on-one psychosocial interventions strain limited resources. A group format could be useful.

5. Environment:

Strengths

- No major strengths noted.

Weaknesses

- No major weaknesses noted.

Milestones:

Acceptable

Data and Safety Monitoring:

Acceptable

Study Timeline:

Strengths

- Detailed and realistic.

Weaknesses

- No major weaknesses noted.

Protections for Human Subjects:

Acceptable Risks and/or Adequate Protections

Data and Safety Monitoring Plan (Applicable for Clinical Trials Only):

- Acceptable

Inclusion Plans:
- Sex/Gender: Distribution justified scientifically.
- Race/Ethnicity: Distribution justified scientifically.
- For NIH-Defined Phase III trials, Plans for valid design and analysis: Not Applicable.
- Inclusion/Exclusion Based on Age: Distribution justified scientifically.

Vertebrate Animals:
Not Applicable (No Vertebrate Animals)

Biohazards:
Not Applicable (No Biohazards)

Resubmission:
Acceptable

Resource Sharing Plans:
Unacceptable

Budget and Period of Support:
Recommend as Requested

CRITIQUE 3

Significance: 2
Investigator(s): 2
Innovation: 3
Approach: 4
Environment: 2

Overall Impact: This is a resubmission to establish feasibility of a multi-site trial of a mind-body treatment (TOR) for orthopedic patients reporting elevated levels of pain anxiety and pain catastrophizing following injury who have been shown to be at greater risk of developing persistent pain and disability, and their downstream effects on function and public health. There are no existing psychosocial programs for these patients. This top-of-the-cliff approach is innovative, and if found deliverable and effective, the program would be a significant addition to the longer-term efficacy of orthopedic treatments following injury. Enthusiasm for the trial is diminished by lack of evidence for the four-session program – reduced from an established 6-session program – given the team's published data referring to a promising 60-second mindfulness trial for orthopedic patients. Given the time and circumstantial constraints of patients, it is important that a program such as this be no longer than is necessary to achieve its clinical goals.

1. Significance:
Strengths
- There are no exiting psychosocial programs for orthopedic patients with these features.
- This is a thoughtful top-of-the-cliff approach that if found deliverable and effective, would provide a program that was a significant addition to the efficacy of orthopedic treatments following injury.
- The scientific rationale for the program and its features is well thought through.
- There is good preliminary clinical data for the program and its delivery, but important features of the program need further testing to lead to changes in clinical practice.

Weaknesses
- The clinical significance of the program is diminished by lack of rationale for the necessity of four sessions for the proposed outcomes. The application refers to a promising 60-second mindfulness intervention trialed in an orthopedic clinic. It's a big jump in time demand from that to a four-session video program. No testing of rationale is given for the decisions that led to the change.

2. Investigator(s):
Strengths
- This is an excellent team of established and successful investigators with complementary skills to accomplish the Aims of the project.
- There appears to be investigators at each of the proposed sites.

Weaknesses
- No major weaknesses noted.

3. Innovation:
Strengths
- This pre-emptive approach to treatment, before pain becomes established is very innovative and deserving of investigation as it its effects.
- It describes thoughtful approaches to barriers of successful completion.

Weaknesses
- The use of a usual care control it is not particularly innovative.

4. Approach:
Strengths
- Potential barriers and problems are well covered and planned for.
- The approach is generally well thought out and described.

Weaknesses
- It is unclear how the videos are integrated with the therapist. Are they on a spilt screen? How does the session proceed? Do the therapist and patient watch the video together, with the therapist taking questions or complementing the video instructions?
- Will some of these patients be recovering from surgery? Could this affect outcomes?
- A justification for usual care control is provided, yet it restricts opportunity to study the length of treatment needed for clinical outcomes. If the four-session video was compared to say a two-session video, it could provide valuable information about clinical feasibility.
- It is unclear how the project will approach reluctant physicians. Given that 50% of the physicians in the pilot study declined to cooperate, there needs to be some explicit plan about what if anything might change their minds about the utility of such a program. A focus group may not be the best approach to these people.
- Since the blessing of the physician was found to be important to participation, the degree of enthusiasm of each referring physician for the program could be assessed and examined in terms of outcomes.

5. Environment:
Strengths
- This is an excellent environment.
- The proposed sites provide an opportunity for a more diverse sample of patients and referring physicians.

Weaknesses
- No major weaknesses noted.

Milestones:

Acceptable

Data and Safety Monitoring:
Acceptable

Study Timeline:
Strengths
- Timeline appears reasonable, even though in such a feasibility study the unexpected can be expected.

Weaknesses
- No major weaknesses noted.

Protections for Human Subjects:
Acceptable Risks and/or Adequate Protections
Data and Safety Monitoring Plan (Applicable for Clinical Trials Only):
- Acceptable

Inclusion Plans:
- Sex/Gender: Distribution justified scientifically.
- Race/Ethnicity: Distribution justified scientifically.
- For NIH-Defined Phase III trials, Plans for valid design and analysis: Not Applicable.
- Inclusion/Exclusion Based on Age: Distribution justified scientifically.

Vertebrate Animals:
Not Applicable (No Vertebrate Animals)

Biohazards:
Not Applicable (No Biohazards)

Resubmission:
Acceptable

Resource Sharing Plans:
Unacceptable

Budget and Period of Support:
Recommend as Requested

CRITIQUE 4

Overall Impact: The study addresses psychosocial determinants of progression to chronic pain after a traumatic orthopedic injury using an intervention delivered via telehealth. The intervention appears to be a multicomponent one based on pain education, cognitive-behavioral approaches, and mind-body practices. The project provides a clear rationale for the importance of the work and present data from relevant preliminary studies. The team includes psychologists, orthopedic surgeons, and an experienced biostatistician. A health psychologist with experience in qualitative research and implementation has been added to the team since the first submission. The application is responsive to previous reviews. The project plans two Aims: 1) a formative study to assess implementation barriers using an implementation science framework (Proctor's) designed to identify barriers and facilitators to implementation; and 2) a randomized, multisite feasibility study. Data collection for the first Aim will consist of focus groups with surgeons and other clinical staff, reportedly using a focus group guide

based on the implementation framework. Although multiple terms from the framework are mentioned, the questions to be included in the focus group guide are not presented. Similarly, although transcribed focus group recordings will be analyzed with “thematic coding” following the framework, the specifics of the qualitative analysis are unclear. Alternatives to focus groups for busy clinicians are not discussed. Rationales for choices for inclusion/exclusion criteria and measures are presented. Aim 2 includes a quantitative analysis of feasibility benchmarks. The sample size calculation within the approach is unclear, but a statistical power and design section is also provided. This section provides adequate discussions of information on treatment assignment, handling of missing data, and sample sizes. The calculations in this section (minimum proportions to achieve benchmarks) are reasonable (and reproducible). The randomized trial will result in multiple feasibility and acceptability measures for which there are clear benchmarks. The trial will also collect patient outcome data before and after the intervention and at 3 months. They plan to report means, standard deviations, and percent change as well as variability estimates. Pre-post effect size measures and their applicability to future studies are not described. In summary, this is a strong resubmission application addressing a reasonably important health condition from a very good investigative team. The resubmission application contains a few lingering addressable flaws.

Milestones:
Acceptable

- Focus groups with medical staff may not be feasible even if encouraged by physician champions. Alternative strategies should be explored. It is unclear how it is possible to offer CME credits for participating in a focus group. A focus on quality improvement, as the project suggests, along with brief telephone interviews with clinicians, may be more successful. Overall milestones are realistic.

THE FOLLOWING SECTIONS WERE PREPARED BY THE SCIENTIFIC REVIEW OFFICER TO SUMMARIZE THE OUTCOME OF DISCUSSIONS OF THE REVIEW COMMITTEE, OR REVIEWERS’ WRITTEN CRITIQUES, ON THE FOLLOWING ISSUES:

PROTECTION OF HUMAN SUBJECTS: ACCEPTABLE

INCLUSION OF WOMEN PLAN: ACCEPTABLE

INCLUSION OF MINORITIES PLAN: ACCEPTABLE

INCLUSION ACROSS THE LIFESPAN PLAN: ACCEPTABLE

COMMITTEE BUDGET RECOMMENDATIONS: The budget was recommended as requested.

Footnotes for 1 U01 AT010462-01A1; PI Name: Vranceanu, Ana-Maria

NIH has modified its policy regarding the receipt of resubmissions (amended applications). See Guide Notice NOT-OD-14-074 at http://grants.nih.gov/grants/guide/notice-files/NOT-OD-14-074.html. The impact/priority score is calculated after discussion of an application by

averaging the overall scores (1-9) given by all voting reviewers on the committee and multiplying by 10. The criterion scores are submitted prior to the meeting by the individual reviewers assigned to an application, and are not discussed specifically at the review meeting or calculated into the overall impact score. Some applications also receive a percentile ranking. For details on the review process, see http://grants.nih.gov/grants/peer_review_process.htm#scoring.

MEETING ROSTER

The roster for this review meeting is displayed as an aggregated roster that includes reviewers from multiple AT Special Emphasis Panels Meetings
for the 2020/01 council round.
This roster for AT is available at:
http://public.era.nih.gov/pubroster/Reports?DOCTYPE=SEP&DESFORMAT=PDF&AGENDA_SEQ_NUM_P=381978
